# A global classification of coastal flood hazard climates associated with large-scale oceanographic forcing

**DOI:** 10.1038/s41598-017-05090-w

**Published:** 2017-07-11

**Authors:** Ana Rueda, Sean Vitousek, Paula Camus, Antonio Tomás, Antonio Espejo, Inigo J. Losada, Patrick L. Barnard, Li H. Erikson, Peter Ruggiero, Borja G. Reguero, Fernando J. Mendez

**Affiliations:** 1 Surf & Surge Research Group. Dpto Ciencias y Tecnicas del Agua y del Medio Ambiente, Universidad de Cantabria, Santander, Spain; 2 Department of Civil and Materials Engineering, University of Illinois at Chicago, Chicago, IL USA; 3 Environmental Hydraulics Institute, IHCantabria, Universidad de Cantabria, Santander, Spain; 4 Pacific Coastal and Marine Science Center, United States Geological Survey, Santa Cruz, CA USA; 5 College of Earth, Ocean, and Atmospheric Sciences, Oregon State University, Corvallis, Oregon USA; 6 Institute of Marine Sciences, University of California, Santa Cruz and The Nature Conservancy, USA

## Abstract

Coastal communities throughout the world are exposed to numerous and increasing threats, such as coastal flooding and erosion, saltwater intrusion and wetland degradation. Here, we present the first global-scale analysis of the main drivers of coastal flooding due to large-scale oceanographic factors. Given the large dimensionality of the problem (e.g. spatiotemporal variability in flood magnitude and the relative influence of waves, tides and surge levels), we have performed a computer-based classification to identify geographical areas with homogeneous climates. Results show that 75% of coastal regions around the globe have the potential for very large flooding events with low probabilities (unbounded tails), 82% are tide-dominated, and almost 49% are highly susceptible to increases in flooding frequency due to sea-level rise.

## Introduction

For more than a century, attempts have been made to classify the entirety of the terrestrial earth into distinct climate types, e.g. the Köppen–Geiger climate classification system^1^. Recently, this endeavor includes the development of improved classification algorithms and their application to climate change^2^. While terrestrial climate types have received significant attention, the definition of oceanic climate types remains undeveloped.

Coastal flooding is the dynamic interaction of a variety of oceanographic processes (e.g. waves, tides and surge levels) and local topographic characteristics. Prior studies of global coastal flooding exposure estimate that about 10 million people per year might experience flooding^3^ under present day conditions, but this prior work does not consider all the significant components of flooding levels, notably waves. In this paper, we analyze the oceanographic characteristics responsible for coastal flood hazards and obtain a preliminary global classification of so-called coastal flood hazard climates, based on the joint influence of astronomical tides, storm surges, and wave-induced elevated water levels. Local topographic characteristics, on the other hand, are not considered in the present classification. In this work, we apply the total water level (TWL), the sum of the astronomical tide (AT), storm surge (SS), and wave setup (WS), referenced to mean sea level (MSL), as a proxy to represent the maximum potential flood hazard, while recognizing that local topographic characteristics influence the site-specific exposure to this hazard. Classifying coastal hazards into representative types is important for understanding regional spatial variability, developing best practices for flood protection, and assessing impacts to habitats and infrastructure from increased surface and groundwater flooding due to sea-level rise^4–10^, among other reasons.

Recent work has explored the variability of different components of TWL (e.g., globally, for the astronomical tide^11^, extreme sea levels^12, 13^, and storm surge^14^ and, regionally, for Latin America and the Caribbean^8^ and the Gulf of Guinea^15^). However, a consistent, global coastal flood hazard classification based on the spatiotemporal variability of TWL remains undefined.

In this work, we obtain a global-scale classification of the primary oceanographic sources of coastal flooding by searching for spatially homogeneous patterns of six physical parameters related to TWL with an automated algorithm. The six parameters that define the coastal flood hazard climates are the three parameters of the generalized extreme value (GEV) distribution of the annual maxima of TWL, the (1) location, (2) scale and (3) shape parameters, as well as the relative contribution of (4) AT, (5) SS, and (6) WS. In the following section, we discuss the six parameters of the classification and the data sources used to derive them.

## Data and Methods

### Data Sources

Hourly time series of astronomic tide (AT) are computed from 13 harmonic constituents provided by the TPXO tidal inversion model^16^, with native resolution of 0.25° × 0.25°. To estimate wave setup, we use an empirical formulation^17^ given by WS = 0.035·β·(Hs·L_0_)^0.5^, where Hs is the significant wave height, L_0_ is the wavelength, both derived from a global hindcast of 1.5° × 1° spatial resolution^18^, and β is the beach slope estimated based on an empirical relationship^19^ where beach slope is estimated as a function of breaking wave heights and periods following the procedure defined in the Supplementary Information. Six-hourly storm surge (SS) levels on a regular grid of 0.25° × 0.25° are produced from the MOG2D model^20^ from Legos and distributed by Aviso, with support from CNES (http://www.aviso.altimetry.fr/). The original data sources are interpolated (using the nearest neighbor approach) to synoptic, hourly datasets with global 1° × 1° spatial resolution. We build a multi-decadal (1992–2013) hourly time series of TWL, composed of the sum of astronomical tide level (AT), surge level (SS), and wave setup (WS), i.e. TWL(t) = AT(t) + SS(t) + WS(t), on a 1° × 1° global grid, relative to mean sea level (MSL). Although WS is not relevant outside the surf zone, it has been defined globally as a proxy for wave-induced contributions to coastal flooding, and to provide information for island communities that are poorly resolved in the global grid. Point-source flooding events, e.g. hurricanes and tsunamis, and sea-level anomalies, e.g. associated with El Nino-Southern Oscillation (ENSO)^21^, are not included on the current water-level data set, which only considers high-frequency oceanographic forcing. Further, it should be noted that vertical land motion, while clearly a contributing factor to flood vulnerability for many regions^22^, is not included in this analysis due to the poor resolution of both large and small-scale patterns and rates, with local variability often playing a dominant role^23^.

### Coastal flood hazard components

Using the *r*-largest order statistic method^24^, we fit the top *r* = 10 annual maxima of TWL at each grid point to a Generalized Extreme Value (GEV) distribution, obtaining maximum-likelihood estimates for its three parameters, namely, location µ, scale ψ, and shape ξ. We also calculate the average relative contribution of AT, SS, and WS (denoted *α*AT, *α*SS, *α*WS, respectively) for the top 10 annual maxima of TWL, e.g. *α*AT = sum(AT)/sum(TWL) and conditioned to *α*AT + *α*SS + *α*WS = 1.

Each of the six parameters provides information about a different aspect of the coastal flood hazard climate: µ represents the average value of an annual extreme event; ψ is an indicator of its interannual variability; ξ defines the degree of exceptionality (i.e. a large value of ξ indicates the potential for very large flooding events with low probabilities of occurrence, and a low value indicates bounded distributions); and large values of *α*AT, *α*SS, or *α*WS indicate that flooding is tide, surge, or wave dominated, respectively.

**Figure 1:**
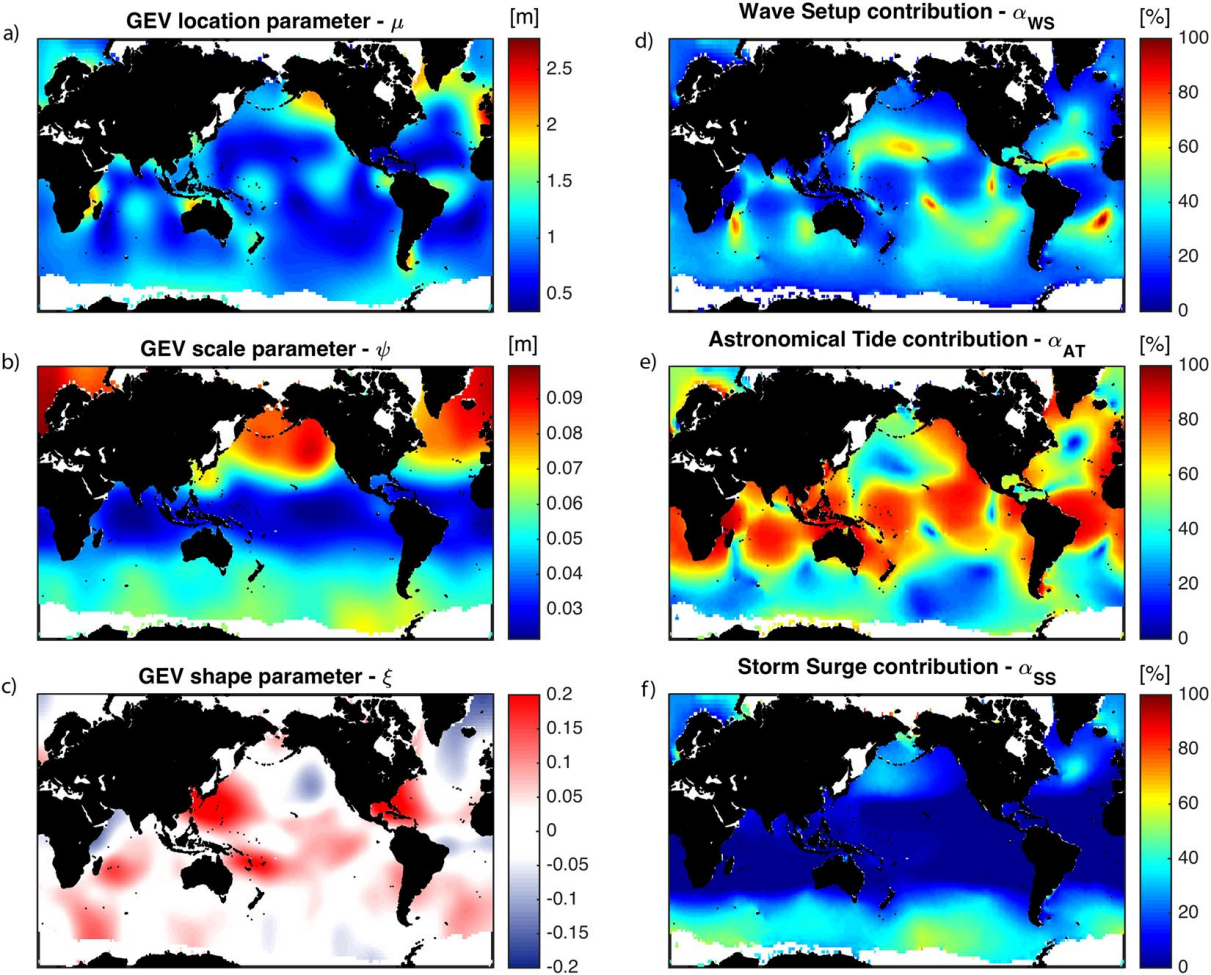
Classification parameters. Global variability of the six classification parameters (µ, ψ, ξ, αAT, αSS, αWS), where µ, ψ, and ξ are the location, scale parameter, and shape parameters of the fitted GEV distribution of TWL, respectively, and αAT, αSS, and αWS are the average relative contribution of the astronomical tide, storm surge, and wave setup to the annual maxima of TWL, respectively. All these maps were created with Matlab 2014b (https://www.mathworks.com/products/matlab/).

Figure 1 shows the global maps of the six parameters. The location parameter, µ, is primarily governed by the amplitude of the tide (see Fig. 1-a and e). The scale parameter, ψ, is highly affected by the meridional gradients in wave energy^25^; ψ is largest in the extratropical (westerlies) regions at high latitudes (particularly in the Northern Hemisphere) and decreasing toward the equator (see Fig. 1-b). Interestingly, the magnitude of ψ also reflects the strong interannual variability of waves in the North Pacific and North Atlantic compared to the slight interannual variability of the Southern Ocean and the weak interannual variability observed in the Tropics^25–27^. Finally, while both the wave and storm surge datasets do not explicitly account for tropical cyclones (TCs) due to the coarse resolution of the atmospheric reanalysis, the positive values of the shape parameter (ξ > 0) in most of the ocean basins, reflects the presence of a few abnormally large extreme water-levels events that dominate the tail of the distribution and coincide with TC prone areas^26^ (see Fig. 1-c). However, in some locations, such as the Indian Subcontinent, the infrequency of TCs results in poor representation of these events using the proposed methodology. To overcome this limitation, stochastic simulations of TCs could extend the population of scarce TC-induced wave and surge datasets^28^. Nevertheless, such an undertaking is still unaffordable on a global scale. Regions with large contributions from storm surge exist predominantly at high-latitudes (particularly in the Southern Ocean) due to the presence of extratropical storms and the low atmospheric pressure fields they induce (see Fig. 1-f). The global average of the relative contribution of storm surge (*α*SS) is only 12%. On the other hand, *α*AT (with a global average of 59%) and *α*WS (with a global average of 29%) are much larger than *α*SS and often play reciprocal roles in determining the primary driver of flooding. For instance, in regions where the tidal range is small due to the proximity of tidal amphidromes, e.g. French Polynesia (see Fig. 1-e), the relative contribution of waves is correspondingly large (see Fig. 1-d).

### Classification algorithm

Thus far, we have defined six parameters to characterize global flood hazard climates. Next, we seek to delineate regions with consistent patterns of the six parameters. The large dimensionality of the problem, a 6-dimensional array X = [µ, ψ, ξ, *α*AT, *α*SS, *α*WS] with 1° × 1° global resolution, precludes hand-drawn classifications. Thus, we apply the objective computer-based classification algorithms described below.

We apply two data-mining algorithms, Self-Organizing Maps (SOM) and K-Means, to the global multivariate array X_i_ = (µ_i_, ψ_i_, ξ_i_, *α*AT_i_, *α*SS_i_, *α*WS_i_), where the subscript represents the i-th grid point. To precondition the classification algorithms, each parameter is first normalized between 0 and 1, e.g. µ* = (µ − min(µ))/(max(µ) − min(µ)). After normalization, the classification is performed on the dimensionless array X_i_* = (µ_i_*, ψ_i_*, ξ_i_*, *α*AT_i_*, *α*SS_i_*, *α*WS_i_*). The Maximum-Dissimilarity-Algorithm (MDA)^29^ is applied, to initialize the clustering algorithms in order to guarantee a good exploration of the 6-dimensional space and avoid the random initialization of the clustering algorithm, which might adversely condition the final results.

**Figure 2:**
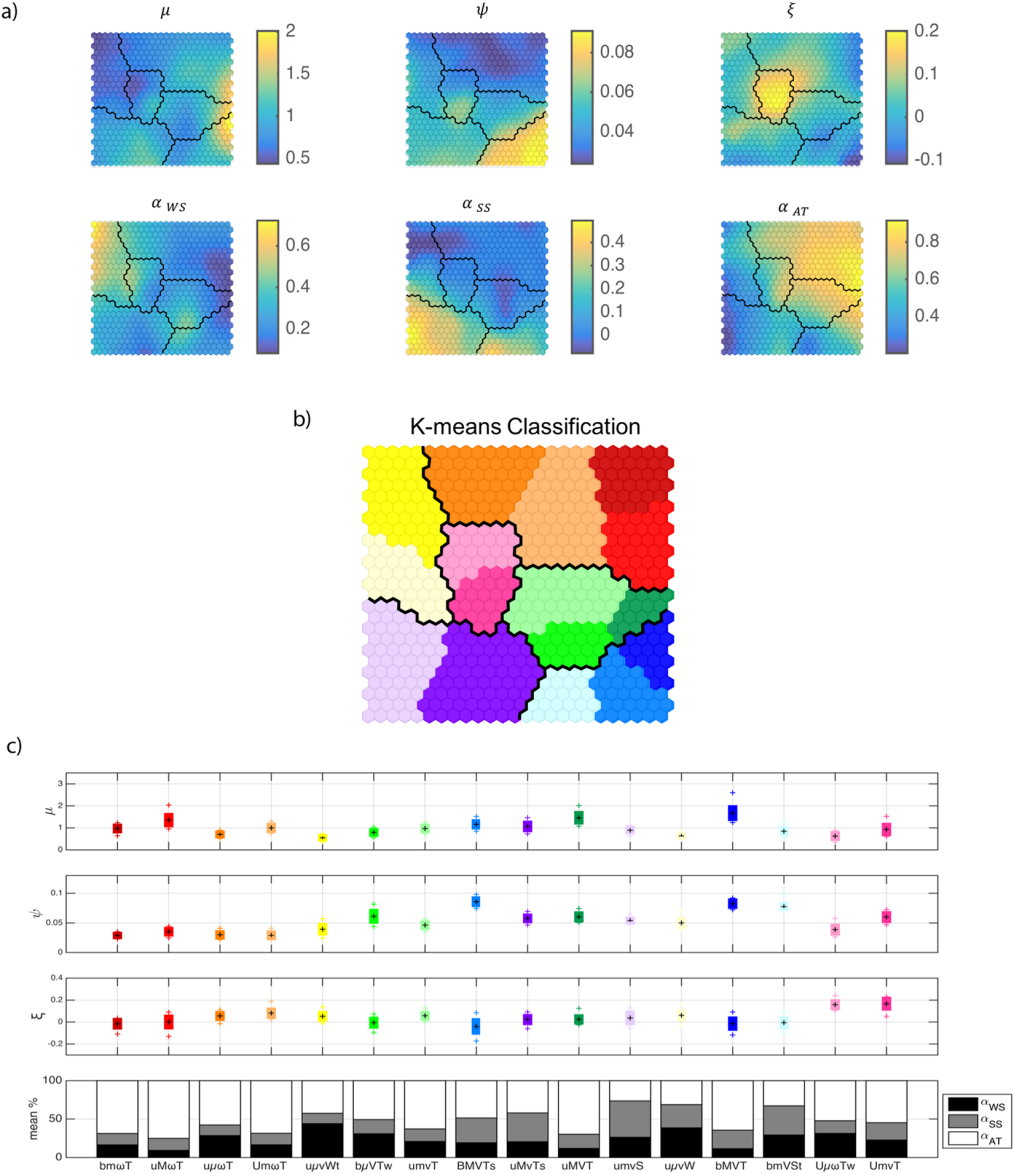
Clusters. (**a**) SOM classification; (**b**) K-Means classification in 16 groups; (**c**) Contribution of each factor.

We first use the SOM algorithm^30^ to obtain a large collection of clusters (NSOM = 25 × 25 = 625). The SOM automatically extracts patterns or clusters of high-dimensional data and projects them onto a bi-dimensional organized lattice (Fig. 2-a). The SOM algorithm makes use of a vicinity criterion to reduce separation between neighboring centroids in the 2D lattice allowing an intuitive visualization of the classification and the correspondence between values of the six parameters.

Next, we apply the K-means algorithm^31^ to find a reduced number (K) of clusters applied to the NSOM centroids of the SOM classification. Here, the optimal number of K = 16 clusters is obtained by applying the Silhouette graphical aid^32^. Finally, we re-dimensionalize the normalized centroids, applying the opposite transformation of the normalization step. Figure 2-b shows the classification of the 16 groups after applying K-means to the SOM centroids. A benefit of the combined SOM/K-means approach is that we can identify similarity between groups: neighboring centroids in the 2D lattice have similar values of the six parameters. For example, the pink groups in the center of the 2D lattice shown in Fig. 2-b are characterized by large values of the shape parameter of the GEV fit (ξ). Figure 2-c shows the mean value of the six parameters associated to each cluster and the variability within each group of the GEV parameters.

## Results

**Figure 3:**
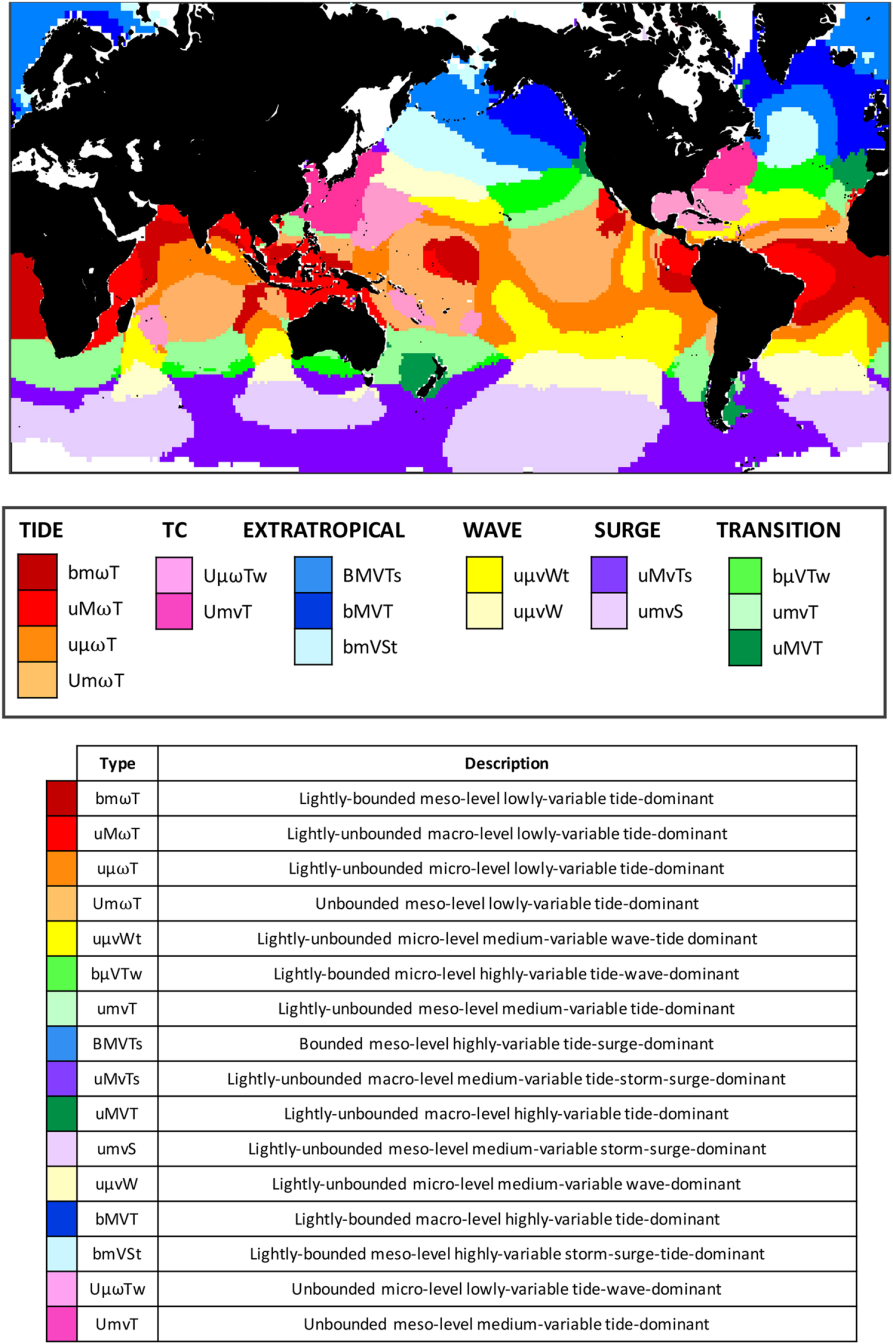
Global coastal flood hazard climates. (Upper panel) Global coastal flood hazard climates; (Lower panel) definition of each climate type. (Table) Type and description of each climate. This map was created with Matlab 2014b (https://www.mathworks.com/products/matlab/).

The geographical distribution of the K = 16 clusters represents coastal flood hazard climates across the global ocean (Fig. 3). Each cluster is defined in terms of the tail of the GEV distribution of TWL, the magnitude of the TWL, the interannual variability, and the predominance of tide, “T”, surge, “S”, or wave, “W”, relative contributions respectively. The thresholds defined for each parameter of the GEV are summarized in Table 1.

**Table 1:** Thresholds for the GEV parameters. Threshold selection to obtain discrete categories according to the location, scale and shape parameters of the GEV distribution.

Tail of the GEV (*ξ*)	B	*ξ* ≤ −0.02	*Bounded*
	b	−0.02 ≤ *ξ* < 0	*Lightly-bounded*
	u	0 ≤ *ξ* < 0.06	*Lightly-unbounded*
	U	*ξ* ≥ 0.06	*Unbounded*
Magnitude of the TWL (µ) (in meters)	µ	*μ* ≤ −0.83	*Micro-level*
	m	−0.83 ≤ *μ* < 1.01	*Meso-level*
	M	*μ* ≥ 1.01	*Macro-level*
Interannual variability (ψ) (in meters)	ω	*ψ* ≤ 0.039	*Slightly-variable*
	v	0.039 ≤ *ψ* < 0.05	*Medium-variable*
	V	*ψ* ≥ 0.05	*Highly-variable*

For example, coastal flood climate “bMVT” means “lightly-bounded, macro-level, highly-variable, tide-dominant” this climate can be found on the northern hemisphere, and some coastal areas affected by it are: the northern part of Spain, France, Ireland, England on the Atlantic Ocean and Alaska, British Columbia, and Washington on the Pacific Ocean. These areas experience large waves and high tidal ranges, and therefore reach high values of the TWL index. These areas also exhibit high interannual variability due to the influence of large-scale climate patterns such as the North Atlantic Oscillation (NAO)^33^ or the Pacific Decadal Oscillation (PDO)^34^. On the other hand, a remarkably different climate would be, for example, “uvWt”, namely the “lightly-unbounded micro-level medium-variable waves-tide-dominant” climate, which has the capital letter ‘W’ for the main contribution and a lower case letter “t” for the second contribution when it is larger than 36%. This climate is mainly characterized by the contribution of the waves and can be found in mid-low latitudes such as western Australia or eastern Madagascar.

A benefit of the SOM/K-means classification algorithm is that closely related groups among the K = 16 clusters of flood hazard climates can be easily detected in the 2D SOM lattice in Fig. 2b. These groups, enclosed by thick black lines in Fig. 2-a and b, are defined as:

- TIDE-dominated (*α*AT ≫ *α*SS, *α*WS) flood hazard climates (bm*ω*T, uM*ω*T, uµ*ω*T and Um*ω*T), which are located mainly between the tropics and and are colored in red scale.
- Tropical cyclone (TC) flood hazard climates (Uµ*ω*Tw and UmVT) with *ξ* > 0, which are colored in pink.
- EXTRATROPICAL flood hazard climates (bMVT, BMVTs, bmVSt) with bounded flood levels (*ξ* < 0) and large interannual variability (large ψ), which are located mainly in the northern hemisphere and are colored in blue.
- WAVE-dominated (*α*WS ≫ *α*AT, *α*SS) flood hazard climates (uμvWt, uμvW) colored in yellow.
- SURGE-dominated flood hazard climates (umvS, uMvTs) with non-trivial storm surge components (*α*SS ≫ *α*AT, *α*WS), which are located predominantly in the Southern Ocean and are colored in purple.
- TRANSITION flood hazard climates (uMVT, umvT, bμVTw), which are located at mid-latitudes colored in green.

Some regions of the globe exhibit rapid transitions in flood hazard climate. For example, the Hawaiian Islands are surrounded by the aggregated groups EXTRATROPICAL (north), WAVE (west), TRANSITION (east) and TIDE (south).

It is interesting to note the similarity between the different ocean basins regarding the derived coastal flood hazard climate types. For example, the spatial distribution of climates uMVT (dark green), bMVT(navy blue), bVTw(green), BMVTs(blue), and bmVSt (clear blue) in North Atlantic and North Pacific have a strong resemblance.

The developed classification (Fig. 3) can be used as a graphical guide to the main drivers of coastal flood hazards. For example, if we are interested in the Atlantic Patagonia, Argentina, we observe that (on Fig. 3) it corresponds to the “uMVT” tide-dominated flood hazard climate (dark green), which is unbounded (experiences sporadic extreme events), macro-level (high water levels) and highly-variable (influenced by large-scale patterns). In this case, the flood hazard climate is likely affected by the Antarctic Circumpolar Wave (ACW) system^35^, which exhibits periodicity of 4–5 years and likely leads to its “highly-variable” classification. For further information, we can use Fig. 2 to obtain the representative values of the multidimensional array X = [µ, ψ, ξ, *α*AT, *α*SS, *α*WS] for this particular flood hazard climate.

## Conclusions and Discussion

The ever-increasing concentration of people and development along coast and the fact that over 10 million people experience flooding each year under present-day conditions^3^ motivates the need to understand and quantify the main drivers of coastal flood hazard at a global scale. Here, we aim to classify the physical contributions of tide, surge, and waves as well as the magnitude, frequency, and interannual variability of global coastal flood hazards. We introduce the first objective global classification of coastal flood hazard climates. The classification is based on (a) use of state-of-the-art, multi-decadal historical reconstructions of tides, storm surges, and waves, (b) the GEV parameters of the extreme total water level, a proxy for coastal flooding potential, (c) the relative contribution of each component of TWL, i.e. astronomical tide, storm surge, and wave setup, and (d) the application of automated clustering techniques to find homogeneous groups representing coastal flood hazard climates associated to large-scale oceanographic forcing.

The developed classification provides a consistent methodology for understanding the mechanisms of coastal flood hazard on a global scale. For example, following previous works such as^36, 37^ who found that regions with narrow (ω) and/or bounded (B,b) water-level distributions will experience the greatest impacts due to sea-level rise (SLR), it is possible using this classification to identify those regions which are particularly vulnerable to changes in each driver of coastal flood hazard. The climates most affected by SLR are associated with the “TIDE” group (bmωT, uμωT, uMωT, UmωT), which exhibit narrow water-level distributions (ω) and are located in the tropics. The climate type potentially most affected by SLR is bmωT, the lightly-bounded, meso-level, lowly-variable, tide-dominated climate, which occurs over much of the equatorial Atlantic and North Indian Ocean. Interestingly, many populated cities presently affected by SLR, e.g. Mumbai, Kochi, Grande Vitoria, and Abidjan^38^, fall within this flood hazard climate.

In addition to the uncertainty and limitations associated with the underlying water-level data (such as the one derived from the models’ coarse resolution), other sources of uncertainty arise throughout the analysis. There is uncertainty associated with the extreme value analysis and with the classification itself. Recently, Monte-Carlo methods have been developed to properly characterize the multivariate extreme value distribution for extratropical^4, 39^ and tropical^28^ storms. However, for simplicity, due to the large global-scale of the analysis performed, we have opted to rely on the extreme value distribution of the TWL, which represents a univariate index that accounts for the contemporaneous occurrence of joint events. Between the different extreme value methods available, we have chosen the r-largest method (RLM) of the ten largest values per year (chosen after a sensitivity analysis of the number of maxima per year). The RLM was selected over the annual maxima method (AMM), since by using more data the estimates of extreme values are improved, resulting in narrower confidence intervals^40^. The peak-over-threshold method was dismissed in this work because a different threshold should be chosen for each site, and threshold selection would have introduced additional subjectivity to the analysis due to temporal and spatial water-level variability around the globe. Despite the inherent uncertainty in the estimation of the parameters, we have relied on (expected value) parameter estimates to perform the classification since other classification techniques that account for uncertainty would have been otherwise required^41^. Finally, additional uncertainty is introduced with the classification itself, although this variability within groups is represented in Fig. 2c. We have used an automated and objective classification and relied on graphical aids, such as Silhouette^32^ to find the optimal number of clusters, however, some expert judgment and subjectivity is undoubtedly introduced. Nevertheless, we expect that the classification obtained, while deterministic in its present form, is able to represent the variability of the parameters that influence coastal flood hazard at a global scale. We anticipate that this preliminary classification will be useful to a wide range of end-users as a first approximation of regional differences in coastal flood drivers arising from climatic/oceanographic factors and as a guide coastal hazard assessment for local studies.

In summary, we have developed a classification of worldwide climatic coastal flooding sources based on the best available historical data sets for the past two decades. The proposed methodology informs the main ocean drivers of coastal flooding on a global scale and it is readily applicable to assess present and future coastal flood hazard vulnerability.

## Electronic supplementary material

Supplementary Information
