# Supplementary material for: A global classification of coastal flood hazard climates associated with large-scale oceanographic forcing
Source: Sci Rep. 2017 Jul 11;7:5038. doi: 10.1038/s41598-017-05090-w (PMC5506008; doi:10.1038/s41598-017-05090-w)
Supplement: Supplementary file 1 — Supplementary Information [file 41598_2017_5090_MOESM1_ESM.pdf]

Supplementary information for

A global classification of coastal flooding hazard climates associated with large-scale oceanographic forcing

Ana Rueda *[1], Sean Vitousek[2], Paula Camus[3], Antonio Tomás[3], Antonio Espejo[3], Inigo J. Losada[3], Patrick Barnard[4], Li Erikson[4], Peter Ruggiero[5], Borja G. Reguero[6], Fernando J. Mendez[1]

[1]Dpto Ciencias y Tecnicas del Agua y del Medio Ambiente, Universidad de Cantabria, Santander, Spain
[2]Department of Civil and Materials Engineering, University of Illinois at Chicago, Chicago, IL, USA
[3]Environmental Hydraulics Institute, IHCantabria. Universidad de Cantabria. Santander, Spain
[4]Pacific Coastal and Marine Science Center, United States Geological Survey, Santa Cruz, CA, USA
[5]College of Earth, Ocean, and Atmospheric Sciences, Oregon State University, Corvallis, Oregon, USA
[6]Institute of Marine Sciences, University of California, Santa Cruz and The Nature Conservancy, USA

* Corresponding author, email: ruedaac@unican.es

Data and methods

Estimated time series of TWL on a global grid of resolution 1°x1° are the baseline data of the proposed classification. TWL, defined as the summation of astronomical tide (AT), storm surge (SS) and wave set-up (WS), is posed as an index that represents a proxy for the flooding hazard magnitude and components of coastal flooding drivers due to climatic effects. We have chosen not to include other processes such as wave run-up or vertical land motion on the TWL estimation due to the coarse resolution of the global-scale grid used in the analysis, which does not resolve locally important and highly variable factors such as beach slope. We seek to characterize drivers of coastal flooding due to large-scale oceanographic processes at a variety of time scales, while considering as much variability as possible in the contribution of the different components. Thus, we seek to apply the longest continuous record available. Validated reanalysis products offer long, spatially-homogeneous time series of water-levels and wave parameters. Accordingly, we have used state of the art databases such as the GOW reanalysis [1] to estimate wave contributions, MOG2D-G [2] to account for

large-scale storm surge, and the TPXO tidal inversion model [3] to include the astronomical tidal influence. The three models have been well validated with altimeter and in situ measurements providing reliable data sources for global applications.

The contribution of waves to TWL, namely wave set-up, is estimated based on the empirical formulation [4] given by $WS=0.035\cdot\beta\cdot(Hs\cdot L_0)^{0.5}$, where Hs is the significant wave height, L_0 is the wavelength, both derived from the global wave hindcast GOW [1]. β represents the beach slope which is estimated for every grid cell based on an empirical relationship between breaking wave heights and periods [5].

$$\beta = 0.12\left(\frac{H_b}{T\sqrt{gD}}\right)^{-1/2},$$

where H_b and T are the breaking wave height and period, respectively, (assumed here as the average of the top 1/3 of significant wave heights and corresponding wave periods), g is gravity, and D is the characteristic diameter of sand grains (assumed to be 250 µm to represent fine to medium-grained sand).

Uncertainty analysis

Many sources of uncertainty exist in the global classification. In order to assess how the classification might vary with uncertainty in the parameters, we have applied a Monte Carlo simulation with one hundred realizations of the 6 parameters for each of the centroids of the SOM and performed a matrix of change analysis (Figure 1) which informs the probability of moving into another k-means cluster or remaining in the same cluster based on the Euclidian distance to the centroids. As seen in figure 1c, much larger probabilities correspond to remaining in the same group, and whenever a

change to a different cluster occurs it is to a neighboring cluster. This indicates the robustness of the classification, and that minor adjustments due to uncertainty would mainly occur on the borders between clusters with similar characteristics.

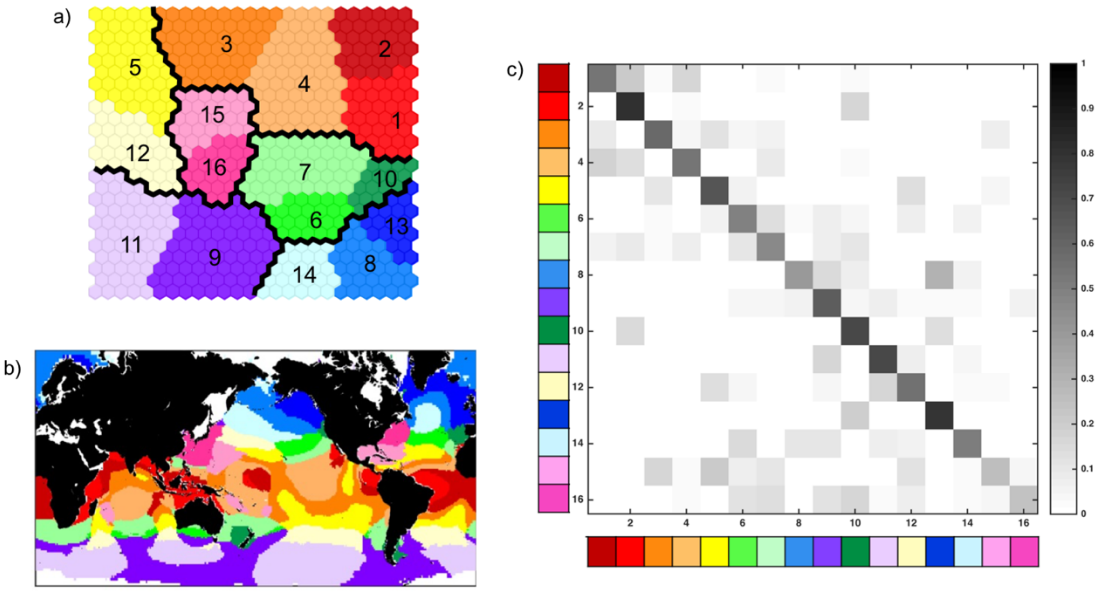

Figure 1. (a) SOM classification. (b) Geographical distribution of the coastal flooding hazard climates. (c) Matrix of probability of change from 100 simulations. This map was created with Matlab 2014b (https://www.mathworks.com/products/matlab/).

References

1. Reguero, B. G., Menéndez, M., Méndez, F. J., Mínguez, R. & Losada, I. J. A Global Ocean Wave (GOW) calibrated reanalysis from 1948 onwards. *Coast. Eng.* **65,** 38–55 (2012).

2. Carrère, L. & Lyard, F. Modeling the barotropic response of the global ocean to atmospheric wind and pressure forcing - comparisons with observations. *Geophys. Res. Lett.* **30,** (2003).

3. Egbert, G. D., Bennett, A. F. & Foreman, M. G. G. TOPEX/POSEIDON tides estimated using a global inverse model. *J. Geophys. Res.* **99,** 24821 (1994).

4. Stockdon, H. F., Holman, R. A., Howd, P. A. & Sallenger, A. H. Empirical parameterization of setup, swash, and runup. *Coast. Eng.* **53,** 573–588 (2006).

5. Sunamura, T. Quantitative predictions of beach-face slopes. *Geol. Soc. Am. Bull.* **95,** 242–245 (1984).
